# Organic Compounds of Arsenic

**Published:** 1909-04-10

**Authors:** 


### THERAPEUTICS AND PHARMACY.

### ORGANIC COMPOUNDS OF ARSENIC.

The limitations to the administration of inorganic preparations of arsenic are considerable owing to intolerance of the drug when the dose is pushed. Even when the pharmacopœial dosage has not been exceeded severe toxic symptoms have sometimes resulted from the administration of liquor arsenicalis, notably in cases of chorea. It is of interest, therefore, to know that arsenic can be given in relatively enormous doses when it is in certain organic combinations discovered in connection with the treatment of sleeping sickness. The drugs so discovered are beginning to be used in other maladies for which arsenic in considerable doses is indicated, notably in pernicious anæmia, leucocythæmia, syphilitic cachexia, and certain skin diseases. The drugs in question are sodium aminarsonate and acetarsonate—the first often sold as Atoxyl and as Soamin, the second as Arsacetin (John Humphrey, *Pharmaceutical Journal*, January, 1909).

### Sodium Aminarsonate.

Sodium aminarsonate is an extremely stable compound, described by Fourneau as the monosodic salt of the anilide of ortho-arsenic acid. As a proprietary product, its trade name is Atoxyl. Neither arsenic nor aniline can be separated from it easily by chemical means. Its aqueous solutions, however, are less stable, pure aniline having been found to be present in solutions that have been exposed to the light for a few days. Boiling decomposes the solutions with liberation of arsenic acid. Atoxyl contains 24 per cent. of arsenium, and yet it is only about one-fortieth as toxic as arsenious acid. This applies to atoxyl itself, of course, and not to an aqueous solution after boiling with liberation of poisonous arsenic acid.

The drug was first employed for anæmia and skin diseases. Then Koch recommended it for the treatment of sleeping sickness. The effect of atoxyl on protozoal infections having been thus demonstrated it was also tried in syphilis, though not with invariable success. Various toxic effects have resulted from big doses, *e.g.* vomiting, diarrhœa, albuminuria, interference with vision, deafness, giddiness, faintness, colic, and weakess of the limbs presumably due to neuritis.

Soamin was introduced as para-aminophenyl arsenate, and was said to differ from atoxyl in the amount of water of crystallisation. It would appear, however, that there is no actual difference in the composition of these two preparations. Soamin has only one-fortieth the toxicity of arsenious acid, and is soluble in about five parts of water.

### Sodium Acetarsonate.

Sodium acetarsonate (proprietary name, Arsacetin) was discovered by Ehrlich, and appears to possess distinct advantages over sodium aminarsonate. The toxic by-effects of atoxyl proving a hindrance to the further development of the therapy of sleeping sickness, syphilis, and so forth, it became important to find a modification of this organic compound of arsenic, which should be less toxic and yet as potent therapeutically. Acetylarsanilate was synthesised by the introduction of an acetyl radicle into the amino group in atoxyl. This compound is free from arsenious and arsenic acids. It is stated to be at least three times less toxic than sodium aminarsonate—whether this be really so or not time will show. We should like it to be understood that we are merely stating what sodium acetarsonate is, without holding any brief in its favour. It is not impossible that in due course it may be found to have effects which detract from its value, and yet another and perhaps a better organic compound of arsenic may be devised. It seems important, meanwhile, however, to understand what the nature of the drug is.

In contrast with sodium aminarsonate, solutions of which seem to be decomposed by boiling, it is stated that solutions of sodium acetarsonate withstand not only boiling, but even heating up to 130° C. for an hour in an autoclave without decomposing. The importance of this from the point of view of sterilisation for injection purposes is obvious; moreover it obviates the necessity for making up fresh solutions daily.

Neisser (*Deutsche Med. Wochenschrift*, No. 35, 1908) is quoted by Mr. Humphrey (*loc. cit.*); he tried arsacetin extensively both on animals and human beings, and reports:—

(1) That the preparation is certainly far less toxic than atoxyl or soamin, both healthy and diseased animals tolerating very much larger doses of arsacetin than of atoxyl; (2) that as far as it is possible to make any comparison as to the remedial action on syphilis the results are in favour of arsacetin; (3) that no decomposition of any kind could be detected in the solution, even when it had been stored for a long time; (4) that boiling daily does not seem to alter the solution.

It must not be forgotten that the results obtained with these drugs in the treatment of syphilis are so far extremely disappointing; nor has any case of sleeping sickness been definitely cured by them, though some improvement is often obtained.

### The Bacteriology of Plasters.

With the object of ascertaining the relative sterility of spread plasters and protective dressings, G. Pinchbeck has conducted a long series of experiments. He communicated the result of his observations to the members of the British Pharmaceutical Conference. He finds that all plasters, unless sterilised, are septic, and that the degree of sterility is diminished by atmospheric pressure. Under these circumstances some effective method of rendering plasters and protective issues sterile is greatly to be desired.

